# PD-L1 status and Immune checkpoint inhibitors in kidney cancer: ignorance, lack of knowledge or both

**DOI:** 10.1007/s00345-021-03624-6

**Published:** 2021-02-13

**Authors:** Hossein Tezval, Jan Hinrich Braesen, Markus A. Kuczyk

**Affiliations:** 1 Department of Urology and Urological Oncology, Hannover Medical School, 30625 Hannover, Germany; 2 Institute of Pathology, Hannover Medical School, 30625 Hannover, Germany

Dear Editor,

Development of the immuno-onclogical therapies within the last years raise the need to optimize the management of patients with metatstatic and advanced renal cell carcinoma (RCC). This should include appropriate patient selection and sequencing the therapy based on the available biomarkers such as PD-L1 Status. The new European Association of Urology (EAU) guideline does not recommend a surgical removal of the kidney in advanced and metastatic RCC for the poor-risk patients according to the IMDC criteria. Furthermore, there is no strong data about effective administration of immune checkpoint inhibition (ICI) or tyrosin kinase inhibitors (TKIs) in adjuvant and neo-adjuvant settings.

All main trials (Checkmate 025, Checkmate 214, Keynote 426, Javelin RENAL 101, imMotion 150, imMotion 151) used an immune checkpoint inhibitor (ICI) with or without a TKI. All above-mentioned trials used one of the most popular PD-L1 scoring system namely immune cell score (IC), tumor proportion score (TPS) or combined positive score (CPS) mostly obtained from the primary tumor. In none of all trials details are given about the PDL-1 Status of the metastasis. Surprisingly, all of the main trials used the 1% of positivity as cut off for PD-L1 positivity (PD-L1 >  = 1%) or negativity (PD-L1 < 1%) irrespective of using IC, TPS or CPS. However, Ueda et al. could show that the PD-L1 expression presents no correlation and effect, if not reverse effect on the response rate like what was observed in the checkmate 025 trial [1]. The authors have used at least 4 different antibodies for the PDL-1 staining used in the above-mentioned trials. It is known that the metastases of the RCC show different biology if compared to the primary tumor. One should consider the heterogeneity of the RCC and challenging the reliability of the site of biopsy and area of the evaluation for the PD-L1 scoring under considering the location of the pathological finding regarding the hot or cold tumor region [2]. We are just wondering about the origin of PD-L1 positivity of >  = 1%, whereas any positivity of PD-L1 in more than 63% and a PD-L1 score up to 50% in some tested specimens has been reported (Javelin RENAL 101 trial).

The synergistic effect of VEGF Blockade in combination therapy with PDL-1 inhibitors on tumor progression should be considered while the PD-L1 expression is accompanied by *VHL* inactivation [3].

There is very limited data about the binding affinity and selectivity of the currently known ligands and receptors such as PD-1, PD-L1 und PD-L2. The next concern is a high probability of existing but yet not discovered other PD ligands and receptors and their possible pharmacological interactions.

There are very limited studies which compared the primary lesions and the metastasis in different organs. Only Zhang et al. could show a different expression of PD-1 expression level between primary lesions of the kidney and brain metastasis in 163 patients with metastasized RCC. PD-L1 expression showed a slight preference in the lung and lymph nodes if compared to the primary tumor. While PD-L2 could be detected in bone metastasis, the primary kidney tumor showed less expression [4].

We should not forget to notice that unfortunately nobody is interested in the behavior of the PD-L1 system in other histopathological entities of RCC which after all represents more than one-fourth of the kidney cancers. Moreover, in all phase 3 trials mentioned above—irrespective to the PDL-1 expression—patients who were believed to be negative in PD-L 1 expression showed a reasonable clinical response to ICI therapy.

All together we can conclude that the available PD-L status with a cut off of 1% expression under the above-mentioned conditions used by major trials is far from a predictive accuracy. Therefore, we are not surprised that the immune checkpoint inhibitors are being used clinically and the supposed PD-L1 scoring cannot play a role for this form of therapy. Practically, we use ICI as a directed therapy and in the same time we fail to care for the target. In our opinion, there are lots of questions about the PD-L status in the primary tumor and its metastases. As long as we do not know about the expression profile of the metastases in a standardized protocol and scoring system for PD-L1, nobody can give any statement about the standing or uselessness of the PD-L-Status in advanced RCC.
